# Integrative Organelle-Based Functional Proteomics: In Silico Prediction of Impaired Functional Annotations in *SACS* KO Cell Model

**DOI:** 10.3390/biom12081024

**Published:** 2022-07-24

**Authors:** Federica Morani, Stefano Doccini, Daniele Galatolo, Francesco Pezzini, Rabah Soliymani, Alessandro Simonati, Maciej M. Lalowski, Federica Gemignani, Filippo M. Santorelli

**Affiliations:** 1 Department of Biology, University of Pisa, 56126 Pisa, Italy; federica.morani@biologia.unipi.it (F.M.); federica.gemignani@unipi.it (F.G.); 2 Molecular Medicine for Neurodegenerative and Neuromuscular Diseases Unit—IRCCS Stella Maris, 56128 Pisa, Italy; stefano.doccini@fsm.unipi.it (S.D.); daniele.galatolo@fsm.unipi.it (D.G.); 3 Department of Surgery, Dentistry, Paediatrics and Gynaecology, University of Verona, 37129 Verona, Italy; francesco.pezzini@univr.it (F.P.); alessandro.simonati@univr.it (A.S.); 4 HiLIFE, Meilahti Clinical Proteomics Core Facility, Faculty of Medicine, University of Helsinki, FI-00014 Helsinki, Finland; rabah.soliymani@helsinki.fi (R.S.); maciej.lalowski@helsinki.fi (M.M.L.); 5 Institute of Bioorganic Chemistry, PAS, Department of Biomedical Proteomics, 61-704 Poznań, Poland

**Keywords:** ARSACS, *SACS*, KO models, omics, biomarkers, mitochondria, lysosomes

## Abstract

Autosomal recessive spastic ataxia of Charlevoix-Saguenay (ARSACS) is an inherited neurodegenerative disease characterized by early-onset spasticity in the lower limbs, axonal-demyelinating sensorimotor peripheral neuropathy, and cerebellar ataxia. Our understanding of ARSACS (genetic basis, protein function, and disease mechanisms) remains partial. The integrative use of organelle-based quantitative proteomics and whole-genome analysis proposed in the present study allowed identifying the affected disease-specific pathways, upstream regulators, and biological functions related to ARSACS, which exemplify a rationale for the development of improved early diagnostic strategies and alternative treatment options in this rare condition that currently lacks a cure. Our integrated results strengthen the evidence for disease-specific defects related to bioenergetics and protein quality control systems and reinforce the role of dysregulated cytoskeletal organization in the pathogenesis of ARSACS.

## 1. Introduction

Autosomal recessive spastic ataxia of Charlevoix-Saguenay (ARSACS, OMIM 270550) is a rare inherited neurodegenerative disease caused by mutations in *SACS* [1], which encodes sacsin (also called DnaJ homolog subfamily C member 29, DNAJC29), a 520 kDa multi-domain chaperone [2,3] mainly involved in mitochondrial dynamics [4,5], autophagy [6], cytoskeletal intermediate filament assembly and dynamics [7,8], and axonal growth [9]. ARSACS, for which there is no available cure, is characterized by early-onset spasticity in the lower limbs, axonal-demyelinating sensorimotor peripheral neuropathy, and cerebellar ataxia [10,11,12]. Although early onset (2–5 years) is typical, cases with onset in late adolescence or adulthood were also identified [13].

Mitochondrial dysfunction is a crucial feature of ARSACS. Indeed, sacsin is also localized in mitochondria and was demonstrated to be involved in different mitochondrial functions [14]. Mitochondrial dysfunction has been linked, with varying levels of evidence, to Alzheimer’s disease (AD), Parkinson’s disease (PD), amyotrophic lateral sclerosis (ALS), and Huntington’s disease. Mitochondria are the main energy powerhouses of cells; through oxidative phosphorylation, they supply most of the ATP needed to fuel essential neuronal functions. Accordingly, their efficient removal when damaged, through a process of mitophagy, is essential for mitochondrial maintenance and neuronal health [15].

Lysosome dysfunction has been linked to the pathogenesis of several conditions, such as lysosomal storage diseases, AD, frontotemporal dementia, and PD [16,17,18,19,20]. Disrupted lysosome function, trafficking, and maturation are observed in several neurodegenerative diseases [21]. Neurons, given the exceptionally long distance between the cell body and distal ends of neurites, require an efficient transport system to ensure rapid collection and degradation of lysosomal substrates throughout their cytoplasm. Early studies demonstrated that the maturation of endolysosomes is coupled to their retrograde axonal transport [22,23].

We recently showed that a reduced degree of autophagosome aggregation and its fusion with lysosomes in a cellular model of ARSACS due to loss of sacsin resulted in reduced clearance of damaged cellular organelles [6]. Therefore, the crosstalk of mitochondria and lysosomes seems crucial for ARSACS pathogenesis: mitochondrial dysfunction leads to lysosomal impairment due to the accumulation of autophagy products, whereas defective lysosomal function, in turn, triggers mitochondrial defects. For these reasons, expanding the current knowledge of mitochondrial and lysosomal functions in ARSACS may facilitate and accelerate biomarker discovery and thus allow the identification of novel targets related to the disease and therapeutic-related strategies.

In the previous work [6], we generated a whole-genome molecular signature profile of *SACS* knocked-out (KO) cells and identified decreased mitochondrial function associated with increased oxidative stress and impaired autophagic flux as pathways related to an “executioner role” in neuronal cell death. 

In the current work, we performed organelle-specific, label-free, quantitative functional proteomics on isolated mitochondrial and lysosomal fractions. We identified ATP biosynthesis, oxidative stress, protein processing together with filaments organization, synaptic depression, and neuronal cell death as the foremost affected biological processes in *SACS* KO cells compared with control ones. Our findings corroborate the results of a previous study utilizing the RNA-seq approach [6] but also highlight mitochondrial (mtDEP) and lysosomal differentially expressed proteins (lysDEP) as potential candidate biomarkers for ARSACS.

## 2. Materials and Methods

### 2.1. Cell Culture and Treatments

Neuronal-like SH-SY5Y cells, a well-known cellular model for the experimental studies in neurodegenerative diseases [24,25], also used as a model to study ARSACS [4], were kindly donated by Prof. Ciro Isidoro, University of Piemonte Orientale, Novara, Italy. The cells were grown in Eagle’s minimum essential medium mixed in a 1:1 ratio with Ham’s F12 medium (Sigma-Aldrich, St. Louis, MO, USA), supplemented with 10% heat-inactivated FBS, 2 mM L-glutamine, 100 U/mL penicillin, and 100 U/mL streptomycin (all from Euroclone S.p.A., Milan, Italy). Cells were kept at 37 °C in a chamber humidified with 5% CO_2_. An exhaustive characterization of the *SACS* KO transfected SH-SY5Y cell line used in this study was previously reported [6]. In order to assess the overall efficacy of the gene editing approach used, we verified the DNA sequence in the KO cell line by Sanger sequencing, highlighting a premature stop codon in exon 4. Loss of sacsin expression was found in cellular lysates by Western blot analysis by comparing the protein abundance in the parental SH-SY5Y line and empty vector-transfected cells.

The mitochondrial uncoupler carbonyl cyanide 4-trifluoromethoxyphenylhydrazone (FCCP, Sigma-Aldrich, St. Louis, MO, USA) was used at 20 μM concentration for 2 h.

### 2.2. Subcellular Fractionation of Cells for Mitochondrial and Lysosome Enrichment

Isolation of mitochondrial and lysosomal fractions from SH-SY5Y WT and *SACS* KO cell lysates was performed using the Qproteome Mitochondrial Isolation Kit (Qiagen, Hilden, Germany) and the Lysosome Enrichment Kit (Thermo Scientific™, Waltham, MA, USA), respectively, according to the manufacturers’ instructions. About 50 mg of cells obtained from four confluent F75 flasks (harvested without trypsin) were processed for organelle enrichment. The pellets containing mitochondria or lysosomes were solubilized in a lysis buffer containing 7 M urea, 2 M thiourea, 4% CHAPS, and protease inhibitors. The protein concentration was determined using the Bio-Rad protein assay (Bio-Rad Laboratories, Inc., Hercules, CA, USA).

### 2.3. Proteomic Analysis

Ten micrograms of mitochondrial or lysosomal fractions obtained from SH-SY5Y cell lysates were digested using a modified filter-aided sample preparation (FASP) protocol, as described elsewhere [26]. Protein content from three independent preparations for each condition (WT and KO mito/lyso fractions) was measured, and three hundred nanograms of protein digest was utilized for DIA nano-liquid chromatography HDMS^E^ (nine normalized abundance values for each quantified protein identifier, including 3 biological replicates and 3 technical repetitions) [27]. Database searches were carried out against human (release 2019_20429 entries) UniProtKB/SwissProt reviewed database, with an ion accounting algorithm that used parameters described elsewhere [20]. Protein quantitation was performed entirely on non-conflicting protein identifications, using precursor ion intensity data and standardized expression profiles. The proteomics data were submitted to MassIVE, https://massive.ucsd.edu (submitted on 20 December 2021, accession number MSV000088592).

### 2.4. Bioinformatic Analysis and Categorization of Proteomic Data

Differentially expressed proteins (DEPs) were identified on the basis of the number of unique peptides used for label-free quantitation (≥2) at FDR <0.01 and the fold change (FC) from averaged, normalized protein intensities |≥1.5|, utilizing *p* ≤ 0.05 by ANOVA in all comparisons. Protein identifiers (IDs) obtained in HDMS^E^ analysis were further filtered for mitochondrial and lysosomal localization adopting a mitochondrial ranking [20] and Gene Ontology terms (cellular component) with the lysosomal association. Bioinformatic categorization of lysDEP was carried out with Ingenuity Pathway Analysis (IPA™) (Qiagen, Hilden, Germany; IPA Winter Release—December 2020 and Spring Release—April 2022; version 73620684). A *Core Analysis* workflow in IPA was used to interpret the dataset, thus identifying the pathways, upstream regulators, disease, and biological functions that are seen to be the most significantly affected based on differential expression of selected proteins. A ‘‘z-score’’ estimated the predicted activation or inhibition of a given biological function. Only annotations with *p*-values < 0.05 and activation z-scores > 1.5 were considered in the bioinformatic analysis.

In order to identify the most relevant” biomarker” candidates, we performed a biomarker analysis. For this purpose, we filtered the experimental data for species (human), node types, disease categories (neurological, metabolic, inflammatory, or skeletal muscle diseases), and set a detectability threshold in at least blood or plasma, thereby prioritizing the potential candidates based on connection to the disease, detection in body fluids and presence in both the proteomic and transcriptomic datasets.

### 2.5. Oxygen Consumption Rate (OCR) Measurement

Measurements of OCR were performed using a Seahorse XFe24 Extracellular Flux Analyzer (Agilent Technologies, Santa Clara, CA, USA), as reported [6]. According to the manufacturer’s instruction and published data [28], the assay was performed under basal conditions and following the addition of inhibitors to derive several parameters of mitochondrial respiration. Basal respiration was derived by subtracting baseline cellular OCR and non-mitochondrial respiration; the OCR value after oligomycin injection was used to derive ATP-linked respiration (by subtracting the oligomycin rate from baseline cellular OCR) and proton leak respiration (by subtracting non-mitochondrial respiration from the oligomycin rate). Moreover, carbonyl cyanide-p-trifluoromethoxy phenylhydrazone (FCCP) injection served to collapse the inner membrane gradient, allowing to record the maximal rate, and maximal respiratory capacity (derived by subtracting non-mitochondrial respiration from the FCCP rate). Lastly, antimycin A and rotenone were added as inhibitors to shut down mitochondrial function, thus revealing the non-mitochondrial respiration. Mitochondrial reserve capacity was calculated by subtracting basal respiration from maximal respiratory capacity. OCR/ECAR ratio allows for measuring a metabolic phenotype and tracing the metabolic changes [29].

### 2.6. Mitochondrial Oxidative Stress Measurement

Mitochondrial-derived ROS (mtROS) production was detected by staining the cells with MitoSOX™ Red reagent (Invitrogen, Waltham, MA, USA). About 5 × 10^4^ fibroblasts or 1 × 10^5^ SH-SY5Ycells were plated in duplicates in a 24-well plate, and after 24 h of seeding, incubated in the dark at 37 °C for 20 min with 10 μM and 5 μM of the probe, respectively. Negative controls were also included. After staining, the cells were washed, and fluorescence was detected in each sample using a flow cytometry-based method. Cells were analyzed within 10–20 min after completion of MitoSOX staining, avoiding possible nuclear accumulation. In parallel, the cell lines were incubated with antimycin A (20 μM) during the last 15 min of the MitoSOX staining to increase mtROS production. A minimum of 5000 gated events was collected on a BD Biosciences Accuri™ C6 flow cytometer (Becton Dickinson, San Jose, CA, USA). All data were analyzed with BD Accuri™ C6 Plus Analysis software (Becton Dickinson). Measurements were normalized by subtracting the blanks.

### 2.7. Immunofluorescence Analysis

Cells were plated on sterile glass coverslips and processed as in [6]. The following primary antibodies were used: mouse monoclonal anti-p62 (Becton Dickinson; dilution 1:200) and rabbit polyclonal anti-LC3 (Sigma-Aldrich; dilution 1:1000), mouse monoclonal anti-Vimentin (Abcam, dilution 1:100), and rabbit-polyclonal anti-Calreticulin (Thermo-Fisher, dilution 1:50). As secondary antibodies (dilution 1:1000), goat anti-mouse or anti-rabbit antibodies conjugated with AlexaFluor 488 or AlexaFluor 555 dye (Cell Signaling Technology Inc., Danvers, MA, USA) were used. Nuclear chromatin was stained with a fluorescent dye, 4,6-diamidino-2-phenylindole dihydrochloride (DAPI, Sigma-Aldrich), at 5 μg/mL. Images were acquired using a Nikon Ti2-E inverted microscope equipped with a DS-Qi2Mc camera and collected with a Nikon ×60 Plan Apocr λ (NA = 1.40) oil immersion objective, using FITC, TRITC, and DAPI detection filter sets.

For the LC3-p62 colocalization, images were processed using the freely available software ImageJ (version 1.53j), and a threshold-based analysis was performed to reveal the degree of overlap between channels. Colocalization was quantified as the fold change in autophagosomes loaded with cargo (yellow areas) normalized to the green channel area (total cargo signal).

### 2.8. Statistics

All experiments were conducted independently three times, if not specified otherwise. Data are given as average values ± SD. Statistical difference between groups were set at: * *p* < 0.05; ** *p* < 0.01; and *** *p* < 0.001 and evaluated by Mann–Whitney test, if not specified otherwise.

All statistic values were automatically calculated using the software GraphPad software (version 9.0.0, GraphPad Software, San Diego, CA, USA).

## 3. Results

### 3.1. Mitochondrial-Specific Proteome Profile

**Figure 1:**
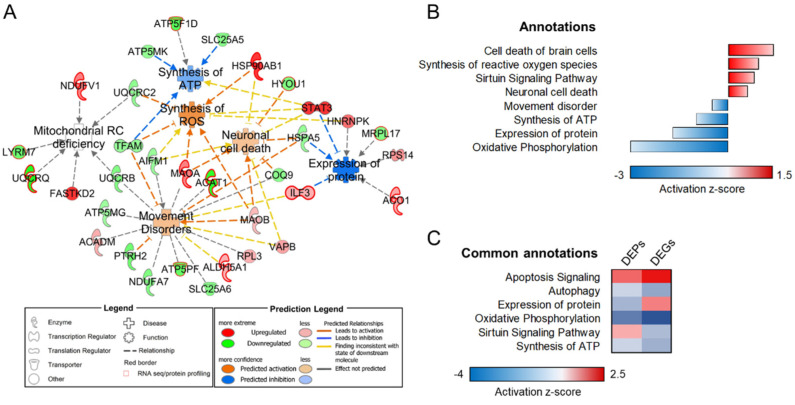
Bioinformatic examination of mitochondria-specific proteome profiles. (**A**) Mitochondria-focused connectivity network displaying differentially abundant proteins (mtDEP) and their major associated diseases and functions. Red-bordered nodes were shared between proteomic and transcriptomic datasets. (**B**) Functional annotations and corresponding activation z-scores in the mitochondrial dataset. Red—up-regulated, blue—downregulated protein abundance. (**C**) Heatmap representation of shared annotations in the mitochondria-focused transcriptome (DEGs, differentially expressed genes) and proteome analyses (DEPs, differentially expressed proteins), sorted according to their z-scores. *n* = 3 in each experimental condition.

Mass spectrometry analysis on mitochondrial fractions resulted in identifying 1443 IDs, of which 618 (42.6% of the total) surpassed the mitochondrial confidence threshold developed by us [20] and reported in Supplementary Table S1. Differentially expressed proteins (DEP) were selected based on the fold change (FC) from averaged, normalized protein intensities |≥1.5|, utilizing *p* ≤ 0.05 by ANOVA in all comparisons. Upon filtering, 55 mtDEP in KO cells were reported (23 up-regulated and 32 down-regulated, Supplementary Table S2). In the bioinformatic categorization, canonical pathways related to *oxidative phosphorylation* (*p*-value 1.26 × 10^−16^; activation z-score = −3.051), whereas among the affected diseases and functions annotations, *synthesis of reactive oxygen species* (*p*-value 3.11 × 10^−3^; activation z-score = 0.943), *expression of proteins* (*p*-value 7.54 × 10^−5^; activation z-score = −1.732), *neuronal cell death* (*p*-value 9.81× 10^−3^; activation z-score = 1.432), and *movement disorder* (*p*-value 1.84 × 10^−5^; activation z-score = −0.503) were pinpointed as the most significantly dysregulated pathways. The connectivity between mtDEP and their associated diseases and functions is portrayed in Figure 1A,B.

### 3.2. Lysosome-Specific Proteomic Profile

**Figure 2:**
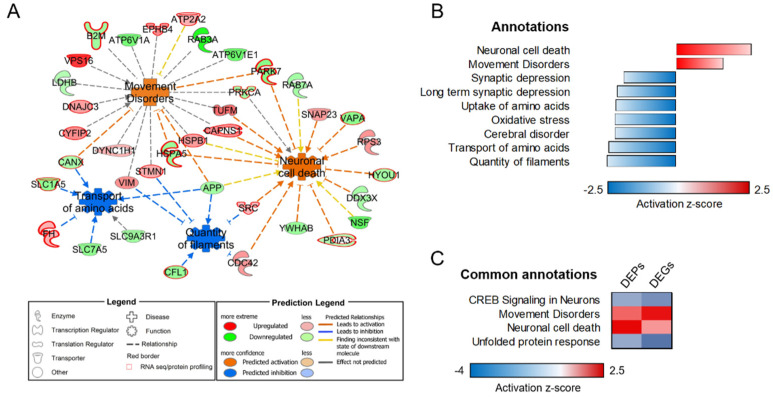
Bioinformatic examination of lysosome-specific proteome profiles. (**A**) Lysosome-specific connectivity network sorted according to predicted, most significantly associated pathway activation/inhibition. Red-bordered nodes represent identifiers shared between proteomic and transcriptomic datasets. (**B**) Functional annotations and linked activation z-scores in ARSACS KO lysosomes. (**C**) Heatmap of shared annotations in lysosome-focused transcriptome (DEGs, differentially expressed genes) and proteome analyses (DEPs, differentially expressed proteins). Red—up-regulated, blue—downregulated protein abundance. *n* = 3 in each experimental condition.

Protein identifiers obtained in HDMS^E^ analysis were filtered for lysosomal localization and ranked based on combined annotations from various lysosomal databases [30,31,32,33] [https://compartments.jensenlab.org/; https://www.proteinatlas.org/] (accessed on 26 April 2022) according to the method reported elsewhere [34], with slight modifications. Mass spectrometry analysis of enriched lysosome fractions identified 1242 IDs, of which 367 (29.5% of the total) were described in at least two lysosomal databases (total score ≥ 3) and thus were assigned with passing confidence for lysosomal localization (see Supplementary Table S1). LysDEP were then selected based on their FC from averaged, normalized protein intensities |≥1.5|, utilizing *p* ≤ 0.05 by ANOVA in all comparisons. By utilizing these filtering criteria, 98 lysDEP in KO cells were accredited (52 up-regulated and 46 down-regulated, Supplementary Table S3). Bioinformatic categorization was performed with the same measures used for mitochondrial counterpart, identifying canonical pathways related to dysregulated *synaptogenesis signaling pathway* (*p*-value 4.17 × 10^−11^), *phagosome maturation* (*p*-value 2.21 × 10^−12^), and decreased *unfolded protein response* (*p*-value 5.02 × 10^−4^, activation z-score = −2.0). Most relevant *diseases and function* annotations highlighted an increased *neuronal cell death* (*p*-value 1.13 × 10^−6^, activation z-score = 2.44) and *movement disorder* (*p*-value 7.38 × 10^−5^, activation z-score = 1.513), together with a decreased *quantity of filaments* (*p*-value 6.71 × 10^−5^, activation z-score = −2.254) and *transport of amino acids* (*p*-value 7.39 × 10^−5^, activation z-score = −2.18). Other annotations related to dysregulated *autophagy, axonal guidance signaling*, *CLEAR signaling, endoplasmic reticulum stress pathway and SNARE signaling* were also highlighted. The links of lysDEP to their associated diseases and functions are presented in the functional connectivity map (Figure 2A,B).

### 3.3. Transcriptome–Proteome Integrative Analysis

We attempted to compare the organelle-specific proteomic dataset with the results obtained in the previous RNA-seq study [6] (raw data are available through Sequence Read Archive; https://www.ncbi.nlm.nih.gov/sra/docs, accession number SRR9302756-61, accessed on March 2021).

**Figure 3:**
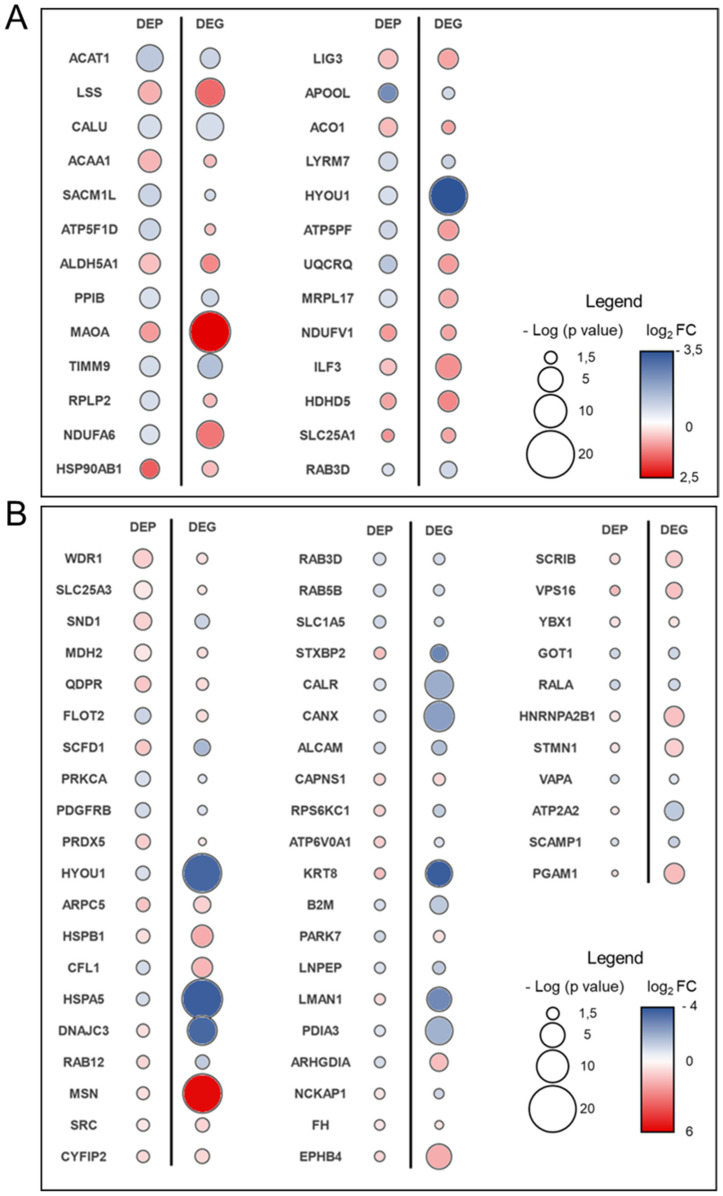
Heat bubble matrix representing the mitochondria-(**A**) and lysosome-(**B**) associated IDs transversally identified as dysregulated in both proteomic and transcriptomic studies. The degree of significance and fold change in differential gene expression/protein abundance are reported as size and color of a bubble, respectively.

Twenty-six out of 55 mtDEP (48%) (Figure 3A, Supplementary Table S4 and red-edged symbols in Figure 1A) were found in the set of DEG, and 20 of them showed the same up- or down-regulation trends. Fifty-one out of 98 lysDEP (52%) (Figure 3B, Supplementary Table S5 and red-edged symbols in Figure 2A) were common to DEG from the same dataset, and 36 displayed the same regulation trend. Conversely, there was a significant number of DEGs/DEPs in both mitochondrial and lysosomal sources that showed different trends of gene expression/protein abundance. Low correlation between transcriptome and proteome supports the view that post-transcriptional modifications play a key role in affecting the amount of active protein after the loss of sacsin [35].

Both subgroups of DEP were subsequently bioinformatically scrutinized to determine the directionality of affected functions. Shared dysregulated organelle-specific functional annotations were revealed by analyzing DEG (from [6]) and both mtDEP and lysDEPs (current study). Similar pathway activity patterns obtained from both types of analyses (global transcriptome and compartmental proteomics profiling) strengthened the involvement of specific cellular pathways as a direct consequence of loss-of sacsin function. Specifically, impaired bioenergetics and defects in autophagy machinery resulted from the integrated mitochondrial analysis (Figure 1C), whereas pathways related to neuronal processes, including homeostasis, metabolism, and survival, emerged from the analysis of the lysosomal compartment (Figure 2C).

In order to prioritize the most relevant biomarker candidates, we surveyed common DEP/DEG datasets (Figure 3 and Supplementary Tables S4 and S5) for the putative links to associated biomarkers. Biomarker analysis pinpointed calreticulin (CALR), endoplasmic reticulum chaperone BiP (HSPA5), hypoxia up-regulated protein 1 (HYOU1), L-lactate dehydrogenase B chain (LDHB), and vimentin (VIM) as putative biomarkers in ARSACS (Table 1).

### 3.4. Experimental Validations to Corroborate In Silico Data

**Figure 4:**
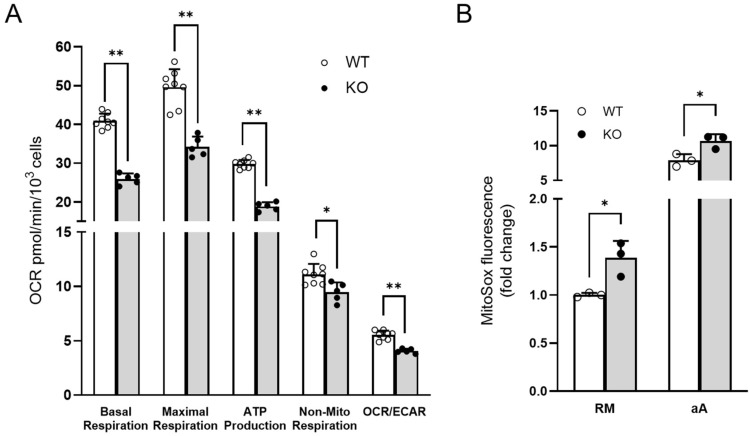
Bioenergetic phenotype in SACS KO cell model of ARSACS. (**A**) Oxygen consumption rate (OCR) was measured in WT and KO cells using the Agilent Seahorse XF Cell Mito Stress Test. The assay was performed under basal conditions and after addition of oligomycin (2 μM), carbonyl cyanide 4-trifluoromethoxyphenylhydrazone (FCCP) (1.5 μM), and rotenone plus antimycin A (1 μM). KO cells showed an impaired energy metabolism compared with WT ones. Data refer to *n* = 8 and *n* = 5 independent measures for WT and KO cells, respectively. * *p* < 0.05; ** *p* < 0.01. (**B**) Oxidative stress was measured by oxidation of MitoSOX fluorescent reagent both in regular conditions (RM = regular medium) and by using antimycin A (aA) as reactive oxygen species (ROS) generator. The production of superoxide by mitochondria was elevated in KO cells. * *p* < 0.05. Each symbol refers to an independent replicate value obtained from a technical triplicate.

In order to functionally validate the involvement of identified dysregulated functions, we focused in our cell model on bioenergetic features, unfolded protein response, and neurofilament dynamics. As predicted in proteomic analyses, OCR measurements validated an overall impairment of energy metabolism, indicated by the reduction in both basal/maximal respiration and ATP production. A significant reduction in OCR to ECAR (extracellular acidification rate) ratio was also detected, suggesting that the metabolic phenotype is more compromised at the OXPHOS level (Figure 4A).

In order to investigate the consequences of an impairment of mitochondrial function determined in the bioinformatic analyses, we further evaluated the changes in the cellular redox state. Mitosox fluorescence showed a 1.5-fold increase in mitochondrial reactive oxygen species (mtROS) production. When ROS production was induced by antimycin A, a similar effect was observed, indicating a change in basal conditions rather than an impairment of detoxification processes (Figure 4B).

**Figure 5:**
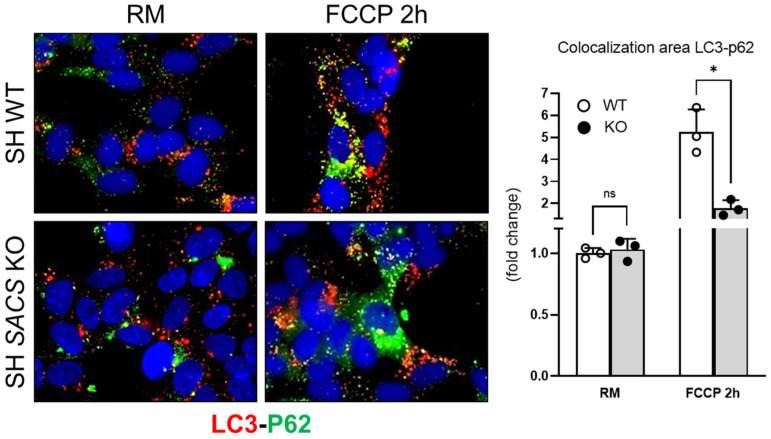
Autophagic flux impairment in SACS KO cells. Representative immunofluorescence images of autophagosomes (LC3, in red) and p62-cargos (in green). The fluorescent dye 4,6-diamidino-2-phenylindole dihydrochloride (DAPI) (in blue) was used as a nuclear stain. WT and sacsin KO cells were analyzed under normal conditions (regular medium—RM) and FCCP treatment (FCCP 2h), and colocalization of LC3 with p62 (yellow areas) was measured as mean ± SD of three replicates, * *p* < 0.05 by two-tailed Student’s *t*-test.

When unfolded proteins accumulate in the endoplasmic reticulum (ER), the unfolded protein response (UPR) works to balance the cellular stress and restore normal proteostasis. Previously, we had demonstrated the involvement of autophagy as the major impaired protein quality control system, excluding proteasome impairment in cells lacking sacsin [6]. Here, through immunofluorescence, we investigated the colocalization of LC3 with p62 in basal conditions and followed mitophagy induction by using the mitochondrial uncoupler FCCP (Figure 5). We observed a reduction in autophagosome–cargo fusion (indicated by yellow dots) during FCCP treatment in *SACS* KO cells, also shown by the colocalization mask. These data, combined with previous observations [6], confirm the impairment of autophagic flux upon *SACS* knockout.

**Figure 6:**
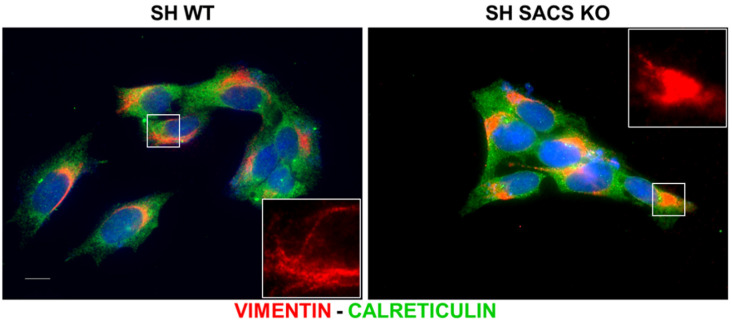
Representative immunofluorescence images for filament network marker, vimentin (red channel) and ER marker, and calreticulin (green channel). Blue channel refers to nuclear staining with DAPI. Scale bar = 10 µm. *n* = 3 independent experiments.

By evaluating vimentin distribution in WT and *SACS* KO cells by immunofluorescence (Figure 6, red channel and Supplementary Figure S1), we confirmed a collapsed intermediate filament network in cells lacking sacsin, as demonstrated earlier [6,7,8]. In parallel, to evaluate the alterations in ER stress pathway, we stained the cells with the ER marker calreticulin (Figure 6, green channel) and did not observe changes in its expression amid WT and *SACS* KO cells. This suggests that knockout of sacsin leads to dysregulation of ER function and cellular homeostasis through different mechanisms rather than altered cellular distribution.

## 4. Discussion

ARSACS remains a challenging condition with limited information on sacsin function in health and disease conditions. The integrative use of quantitative omics offers a possibility to reveal disease mechanisms in ARSACS, candidate biomarkers, and potential new targets for trial readiness, similarly suggested for other neurodegenerative conditions [36,37,38,39].

By leveraging our previous RNA-seq analysis [6] and a more recent aptamer-based targeted approach [25], we observed, for the first time, that loss of sacsin affects neuroinflammation, synaptogenesis, and engulfment of cells, thereby demonstrating the feasibility of multi-dimensional integration of omics data in a rare human condition, an approach familiar more to cancer biology than neurodegeneration. The integrative use of omics facilitated corroboration of a specific defect in the quality control system at the autophagosome–lysosome fusion level reinforced the role of dysregulation in cellular metabolism and intermediate filaments, and allowed identifying novel pathways (i.e., sirtuin signaling; transport and uptake of amino acids) that warrant further research. Obviously, our research approach was not free from limitations, aiming to target specific cellular processes. Nevertheless, it should be considered that the overlap of transcriptomic information with high confidence cellular compartment-specific proteins represents a further step towards identifying disease-specific processes. Moreover, the used filtering strategy facilitated the identification of highly categorized, putative biomarkers that may be worth testing in a clinical setup. Neuronal cells are more sensitive to protein misfolding than non-neuronal cells, in which cell duplications help to prevent the accumulation of unfolded proteins. Protein misfolding has been considered among the initial events underlying neurodegeneration [40,41,42,43], and our new data showing involvement of protein folding quality control via ER calcium-binding proteins bring the mechanisms of ARSACS even closer to the principal of neurodegenerative disorders [44,45]. Identifying molecules that can target quality control systems (UPR/autophagy signaling components) seems critical for the discovery of new therapies for neurodegenerative diseases. CALR, a chaperone protein located in the ER and involved in protein folding quality control and calcium homeostasis, appears as a putative biomarker indicating loss of sacsin, as its expression level was found to be specifically downregulated in lysosomes and mitochondria but not in the whole KO cells (Figure 6, green channel). Further investigations are required to clarify how sacsin dysregulation at the subcellular level could impact the whole cell physiopathology. Apart from its canonical roles in protein folding and calcium homeostasis, CALR also performs roles in neuronal plasticity, synaptic regulation, regulation of gene and protein expression, apoptotic susceptibility, and phagocytosis detrimental to several neurological disorders such as ischemic stroke [46,47,48,49], AD [50], PD [51,52], psychiatric disorders [53,54,55], and ALS [56,57]. Furthermore, ER stress and dysregulation also seem to play a critical role in ataxia-telangiectasia and in the fly model of Friedreich’s ataxia [58]. Thus, it is tempting to speculate that protein folding quality control and calcium homeostasis are additional mechanisms to consider when exploring the possible treatment approaches in ARSACS.

Our data also indicate a tight link between ARSACS and the inflammatory process through microglia activation, in a manner similar to what occurs in common forms of neurodegeneration [59,60]. Through transcriptome–organelle–proteomic data integration analysis, we observed a downregulation in the levels of B2M, a component of class I major histocompatibility complex. B2M is involved in the presentation of peptide antigens to the immune system [61], interferon-gamma-mediated signaling pathway [62], immune-response regulation, negative regulation of neurogenesis, and neuron projection development, and positive regulation of cellular senescence [63]. Of note, we found B2M to be downregulated in a previous targeted assay, where cells were treated with FCCP [64]. Together, the combination of abnormal interferon signature (typical of early-onset neurodevelopmental disorders) and early microglia activation proposes sacsin as a critical factor in both neuronal maturation and neurodegeneration.

Vimentin is a type III intermediate filament protein [65]. According to previous works [6,8], where vimentin was analyzed in ARSACS patient fibroblasts and in cells where sacsin expression was reduced, we observed its abnormal perinuclear accumulation in KO SH-SY5Y cells, indicating a disorganized intermediate filaments pattern that could reflect changes in the cellular distribution of proteostasis machinery. Moreover, we found a common upregulation of the protein in both mitochondria and lysosome profiles. Vimentin can be secreted in extracellular vesicles, including exosomes, found in bodily fluids [66,67]. Soluble vimentin was detected at higher levels in the sera of colon cancer patients, suggesting that it might represent a potential disease biomarker [68]. In combination, these findings provide solid reasons to consider vimentin as a good candidate biomarker for ARSACS.

Although its value as a true biological fluid biomarker needs to be tested, we intend to use the results of our integrative analyses as proof of principle for protein expression screening studies in patients’ blood, starting from those who are already collected in our center. Potential exploitation of a larger group of patients will be possible through networking with international consortia. The use of a relatively large cohort of patients, also at different time points of disease progression or treatment, will offer further applicability to our bioinformatic results.

An additional element of interest emerging from the present work is the possibility of querying the druggability status of candidate proteins. We observed a dozen of potential targets of FDA-approved drugs, including, among others, SRC, B2M, HSP90AB1, MAOA, LSS, ALDH5A1, HSPA5, and vimentin (Supplementary Table S6). This could be a subject for future in vitro screening. Interestingly, SRC, a non-receptor tyrosine kinase that is closely related to tumors, appears to act as a central mediator in multiple signaling pathways, including neuroinflammation, and it protects dopaminergic neurons, improving motor behavior in an MPTP-treated mice PD model [69]. A key role for MTSS1/SRC family kinase dysregulation was also shown in Purkinje neurons survival and ataxia progression in SCA1 and SCA2 mouse models [70]. Inhibition of SRC corrects Purkinje neurons’ basal firing and delays ataxia progression. It is tempting to hypothesize the repurposing of SRC inhibitors for ARSACS treatment. Drugs such as dasatinib, saracatinib, bosutinib, and pyrazolopyrimidines (PP1 and PP2) [71,72], some already in phase II/III clinical trials for brain tumors and AD (see https://clinicaltrials.gov, accessed on 8 June 2022), could be tested for this purpose. Similar speculation could be applied to anthracycline 4’-iodo-4’-deoxydoxorubicin (I-DOX), tested in the treatment of amyloid-related disorders [73,74]. I-DOX inhibits the production of amyloid A and transthyretin and could modulate the expression of B2M [75], another integrated DEPs/DEGs in ARSACS. Moreover, pritumumab binds to the ecto-domain of vimentin on the surface of cancer cells with no off-target effects or not impairing the function of normal vimentin [76,77], thus offering an opportunity for testing in ARSACS models.

In summary, characterization of dysregulated pathways using multiple omics approaches constitutes a suitable strategy for identifying novel drivers of disease progression, as well as targets of novel therapies. The combined use of a transcriptome–organelle–proteomic data integration analysis pinpointing “druggable” targets in ARSACS disease may be the first step towards “omics medicine” in other rare neurodegenerative conditions.

## Figures and Tables

**Table 1:** List of common biomarker candidates.

Accession		Family	Drug(s)	Mitochondrial		Lysosomal		Plasma/Serum
				*p*-Value	Log_2_FC	*p*-Value	Log_2_FC	
CALR	P27797	ER chaperone		1.4 × 10^−4^	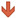 −0.45	1.5 × 10^−5^	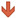 −0.70	x
HSPA5	P11021	enzyme	SHetA2, PAT-SM6	1.2 × 10^−5^	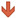 −0.55	6.3 × 10^−7^	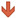 −0.82	x
HYOU1	Q9Y4L1	other		5.7 × 10^−5^	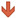 −0.66	7.7 × 10^−8^	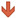 −0.74	x
LDHB	P07195	enzyme		5.7 × 10^−2^	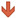 −0.39	3.3 × 10^−4^	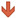 −0.62	x
VIM	P08670	other	pritumumab	9.2 × 10^−2^	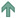 1.11	2.6 × 10^−4^	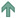 0.93	x

## Data Availability

All data generated or analyzed during this study are included in this article and in [6]. Mass spectrometry data were deposited to the MassIVE database under accession number MSV000088592.

## Supplementary Materials

The following are available online at https://www.mdpi.com/article/10.3390/biom12081024/s1: Supplementary Table S1: Queried databases and criteria employed for compartmental localization of datasets. Supplementary Table S2: Proteomic dataset of mitochondrial DEPs. Supplementary Table S3: Proteomic dataset of lysosomal DEPs. Supplementary Table S4: Mitochondria-associated differentially regulated IDs identified in transcriptomic and proteomic studies. Supplementary Table S5: Lysosomal-associated differentially regulated IDs identified in transcriptomic and proteomic studies. Supplementary Table S6: Druggability of common proteins/genes across the omics studies. Supplementary Figure S1: Representative images of vimentin network in WT and SACS KO neuroblastoma cells.
