# Supplementary material for: Integrative Organelle-Based Functional Proteomics: In Silico Prediction of Impaired Functional Annotations in SACS KO Cell Model
Source: Biomolecules. 2022 Jul 24;12(8):1024. doi: 10.3390/biom12081024 (PMC9331974; doi:10.3390/biom12081024)
Supplement: Supplementary file 1 [file biomolecules-12-01024-s001.zip › biomolecules-1788063-supplementary.pdf]

Supplementary Figure S1

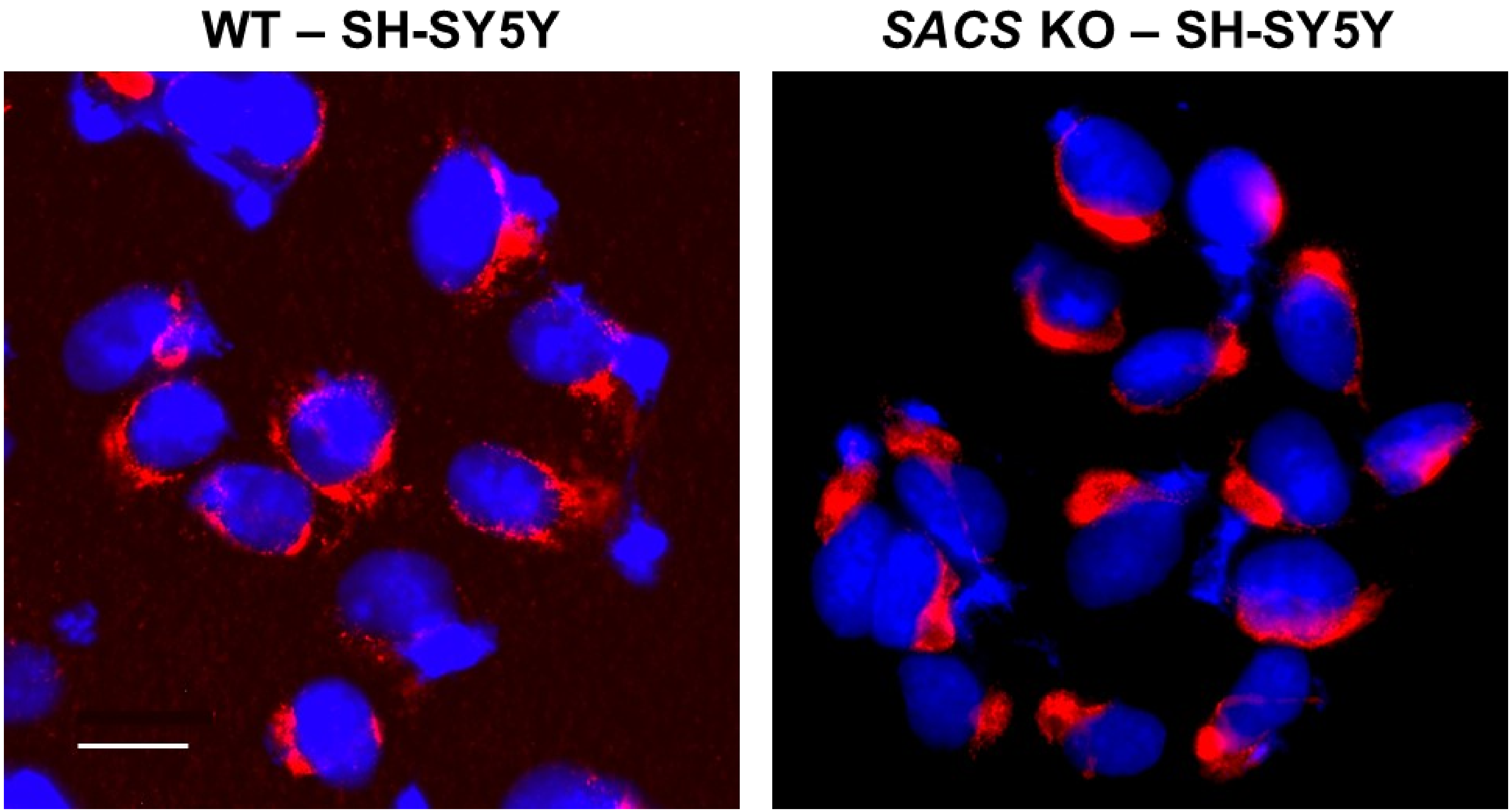

Supplementary figure S1: Representative images of vimentin network (in red) in WT and sacsin KO cells showed a collapsed intermediate filament network in cells lacking sacsin. DAPI (in blue) was used as nuclear stain. Scale bar = 10 µm.
